# Phenotypic and Functional Alterations of Hematopoietic Stem and Progenitor Cells in an In Vitro Leukemia-Induced Microenvironment

**DOI:** 10.3390/ijms18020199

**Published:** 2017-02-14

**Authors:** Jean-Paul Vernot, Ximena Bonilla, Viviana Rodriguez-Pardo, Natalia-Del Pilar Vanegas

**Affiliations:** 1 Cellular and Molecular Physiology, Biomedical Research Institute, Faculty of Medicine, Universidad Nacional de Colombia, Bogotá D.C. 111321, Colombia; xmbonillaf@unal.edu.co (X.B.); npvanegasa@unal.edu.co (N.-D.P.V.); 2 Inmunobiology and Cellular Biology, Faculty of Science, Pontificia Universidad Javeriana, Bogotá D.C. 110231, Colombia; vivianar@javeriana.edu.co

**Keywords:** hematopoietic stem cells, progenitor cells, leukemic niche, cell malfunction, microenvironment, mesenchymal stem cell, proliferation, adhesion, multipotency, REH-conditioned medium

## Abstract

An understanding of the cell interactions occurring in the leukemic microenvironment and their functional consequences for the different cell players has therapeutic relevance. By co-culturing mesenchymal stem cells (MSC) with the REH acute lymphocytic leukemia (ALL) cell line, we have established an in vitro leukemic niche for the functional evaluation of hematopoietic stem/progenitor cells (HSPC, CD34+ cells). We showed that the normal homeostatic control exerted by the MSC over the HSPC is considerably lost in this leukemic microenvironment: HSPC increased their proliferation rate and adhesion to MSC. The adhesion molecules CD54 and CD44 were consequently upregulated in HSPC from the leukemic niche. Consequently, with this adhesive phenotype, HSPC showed less Stromal derived factor-1 (SDF-1)-directed migration. Interestingly, multipotency was severely affected with an important reduction in the absolute count and the percentage of primitive progenitor colonies. It was possible to simulate most of these HSPC alterations by incubation of MSC with a REH-conditioned medium, suggesting that REH soluble factors and their effect on MSC are important for the observed changes. Of note, these HSPC alterations were reproduced when primary leukemic cells from an ALL type B (ALL-B) patient were used to set up the leukemic niche. These results suggest that a general response is induced in the leukemic niche to the detriment of HSPC function and in favor of leukemic cell support. This in vitro leukemic niche could be a valuable tool for the understanding of the molecular events responsible for HSPC functional failure and a useful scenario for therapeutic evaluation.

## 1. Introduction

By finely harmonizing the processes of differentiation and self-renewal, proliferation, and quiescence in hematopoietic stem cells (HSC), the bone marrow (BM) microenvironment is able to generate and maintain appropriate numbers of hematopoietic progenitor cells (HPC) and mature blood/immune cells while maintaining the HSC pool. This microenvironment, or “niche” [1], is assumed to be formed by multiple cell types, with a predominant role played by mesenchymal stem cells (MSC), soluble factors, and extracellular components that could even structure a tailored niche for all stem, progenitor, and immune cells [2,3,4].

Early experiments have also suggested an important role for the microenvironment in the pathogenesis of some human diseases [5], but only recently has it been proven that the microenvironment plays a pivotal role in cancer initiation and progression [6,7]. In fact, mouse models have shown that BM microenvironment alterations are sufficient for malignant hematopoietic transformation [8,9,10,11,12]. Initial work has also shown that BM survival stimuli are essential for clonal expansion of leukemic progenitors [13]. More recently, in chronic myeloid leukemia (CML), it has been shown that protein kinase-C-β-dependent signaling in MSC was necessary for leukemia cell survival [14]. The fact that this signaling is relevant in other hematological malignancies, including acute lymphocytic leukemia (ALL) [14], suggests that a widespread response for leukemogenesis support can be induced in the BM microenvironment, and that this finding could be generally applied for therapeutic interventions. In addition, it has been demonstrated that niche cells play a protective role in CML and ALL in response to chemotherapeutic agents [15,16], implying that by this means survival support and drug resistance could be targeted.

Furthermore, it has recently been shown in transgenic mouse models that leukemic cells alter the niche and normal functioning of stem and progenitor cells (HSPC). Reduced SDF-1 secretion, essential chemokine for HSC homing and retention, and an increase of other chemokines and cytokines by BM stromal cells are responsible for impaired support for HSC and a selective growth advantage for leukemic cells [17,18,19]. Leukemic cells induced an SCF-dependent CD34+ progenitors’ displacement to novel malignant microenvironments [20]. Also, in ALL, leukemic cell dissemination rebuilds a protective microenvironment to assist in survival during primary chemotherapy [16]. Intriguingly, in this study it was shown that the niche components are transient and dynamic, suggesting that the in vivo interplay between leukemic cells and the microenvironment could be much more complex than expected.

In the context of these new findings using mouse models, an in vitro system where the diverse niche components are better defined would be helpful for elucidating the various mechanisms involved in niche remodeling, HSPC functional failure, and leukemic cell survival and resistance to therapy. This system could be a valuable tool for discovering innovative therapies and for the interrogation of in vivo findings. In the present study, we were interested in finding out if we could recreate in vitro the leukemic microenvironment or niche (LN) in order to study HSPC (CD34+ cells) function. Using a short co-culture period of MSC with an ALL-B cell line or leukemic blasts from an ALL-B patient as models of the LN, we were able to show that HSPC in contact with the LN are affected in different ways, partially resembling what has been reported for HSC in vivo. Our in vitro model could be a valuable system for studying the dynamic and evolving interactions between leukemic cells and niche components.

## 2. Results

### 2.1. CD34+ Cells and MSC Isolation and Characterization, and Leukemic Niche (LN) Establishment

We have established an in vitro model of the leukemic niche (LN) by incubation of MSC (Figure S1A,B) with REH cells for three days. After REH cell removal, fresh CD34+ cells (Figure S1C) were added to the LN. CD34+ cells from the same umbilical cord blood (UCB) sample were used to establish the so-called normal niche (NN) with MSC in exactly the same conditions. CD34+ cells characteristics and functional properties were evaluated after three days of co-culture in the LN and the NN. REH cells from the LN were removed by washings thrice with phosphate-buffered saline (PBS) and once with PBS plus Ethylenediaminetetraacetic acid (EDTA), and the remaining REH cell population was evaluated via light microscopy and FACS (usually between 10% and 15%). It was necessary to remove part of the REH cells before CD34+ cells addition, since the overgrowth of REH cells made the evaluation of CD34+ cells almost impossible. Additionally, prolonged MSC-REH co-cultures (>4 days) were not feasible, since REH cells increased their adherence to MSC and were very difficult to remove without damaging MSC cells. After three days of co-culture with CD34+ cells, the amount of REH cells corresponded to near 50% of the cells present in the wells (Figure S2D). In this way, at the end of the incubation period, no overgrowth of the leukemic cells in the LN niche was observed. CD34+ cells could be distinguished from the leukemic cells by their CD34 or CD44 expression (Figure S2A), since REH cells do not express these cell surface markers (Figure S2B) and co-incubation with MSC did not change CD34 or CD44 expression in REH cells upon daily assessment for one week (Figure S3). Alternatively, in some experiments CD34+ were CSFE-labeled before co-culturing with MSC and could then be distinguished easily from REH cells (Figure S2C).

### 2.2. Proliferation, Adhesion, and Migration of CD34+ Cells in the LN

**Figure 1:**
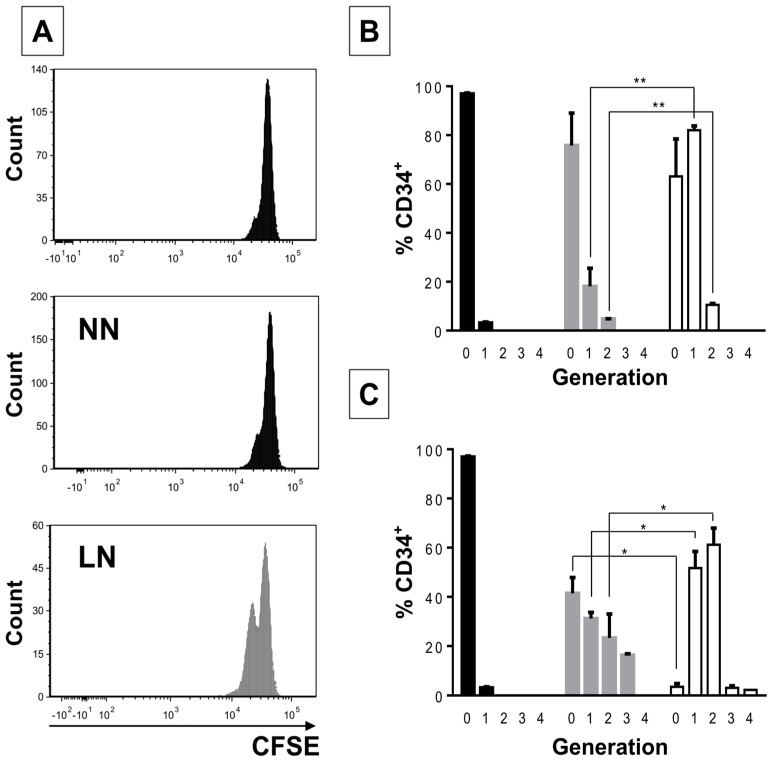

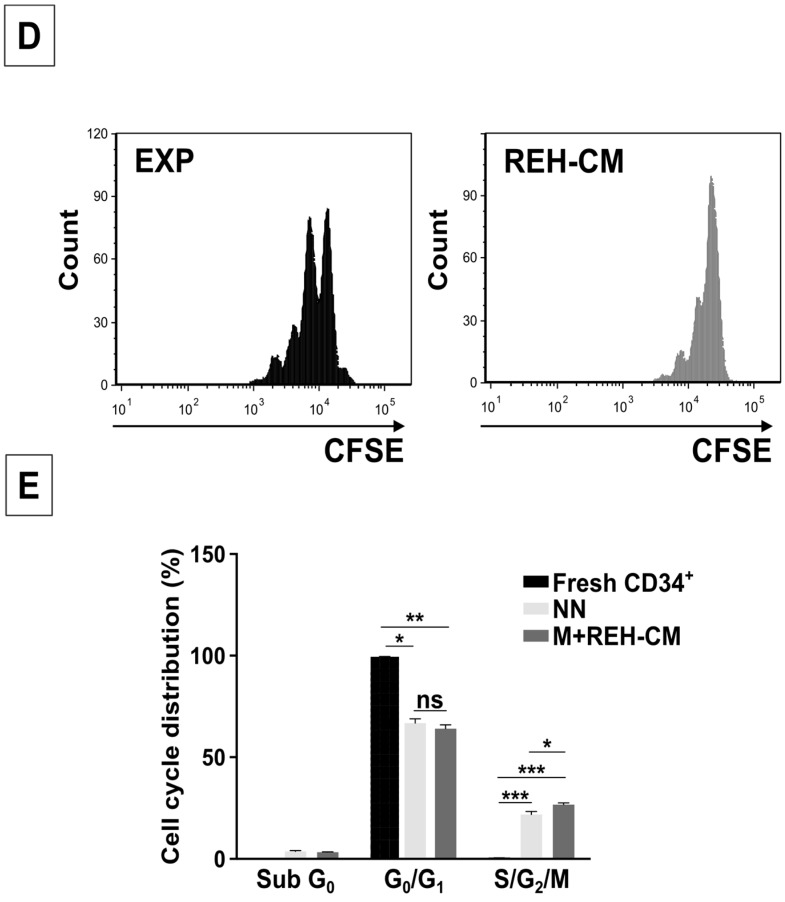
Increased proliferation capacity of CD34+ cells in the LN. (**A**) CD34+ cell proliferation in the NN and the LN was determined by cell staining with CFSE after three days of incubation. The histogram in the upper panel shows CD34+ cells synchronized by serum starvation (FBS removal for two days); (**B**) percentage of CD34+ cells at the corresponding number of cell divisions (0, 1, 2, 3, or 4) in the synchronized cells (black), NN (grey), and the LN (white) after three days of co-culture; (**C**) Percentage of CD34+ cells at the corresponding number of cell divisions (0, 1, 2, 3, or 4) in the synchronized cells (black), NN (grey) and the LN (white) after six days of co-culture; (**D**) CD34+ proliferation evaluated by CFSE staining after three days of culture in cell expansion conditions (FLT-3L, TPO, and SCF, all at 50 ng/mL) (EXP) or incubated with the REH-CM (**E**). Freshly isolated CD34+ or CD34+ from the NN and the M+REH-CM were stained with Hoechst 33342 dye for cell cycle evaluation by flow cytometry (FACSAria™ II, BD Biosciences, San Jose, CA, USA). (ns: non-significant, * *p* < 0.05, ** *p* < 0.01, *** *p* < 0.001). Results shown represent two independent experiments done in duplicates (*n* = 4).

CD34+ cells from the LN exhibited increased proliferation compared to CD34+ cells from the NN (Figure 1A,B). After three days, most (80%) CD34+ cells in the LN had undergone one cell division, while only a minority (20%) had done so in the NN (Figure 1B). This effect was more evident after six days of co-culture (Figure 1C) and was similar to CD34+ cells in expansion cultures, i.e., cells incubated with early cytokines in the absence of MSC (Figure 1D, EXP). Interestingly, the REH-conditioned medium (REH-CM) also induced high CD34+ cell proliferation after three days of stimulation (Figure 1D, REH-CM), closely resembling proliferation induced by early cytokine treatment (Figure 1D, EXP) (four cell generations for cytokine-expanded cells vs. three cell generations for the REH-CM-treated cells). Cell cycle evaluation with Hoechst staining showed also an increase in S/G_2_/M cell populations in CD34+ cells from the M+REH-CM (Figure 1E).

**Figure 2:**
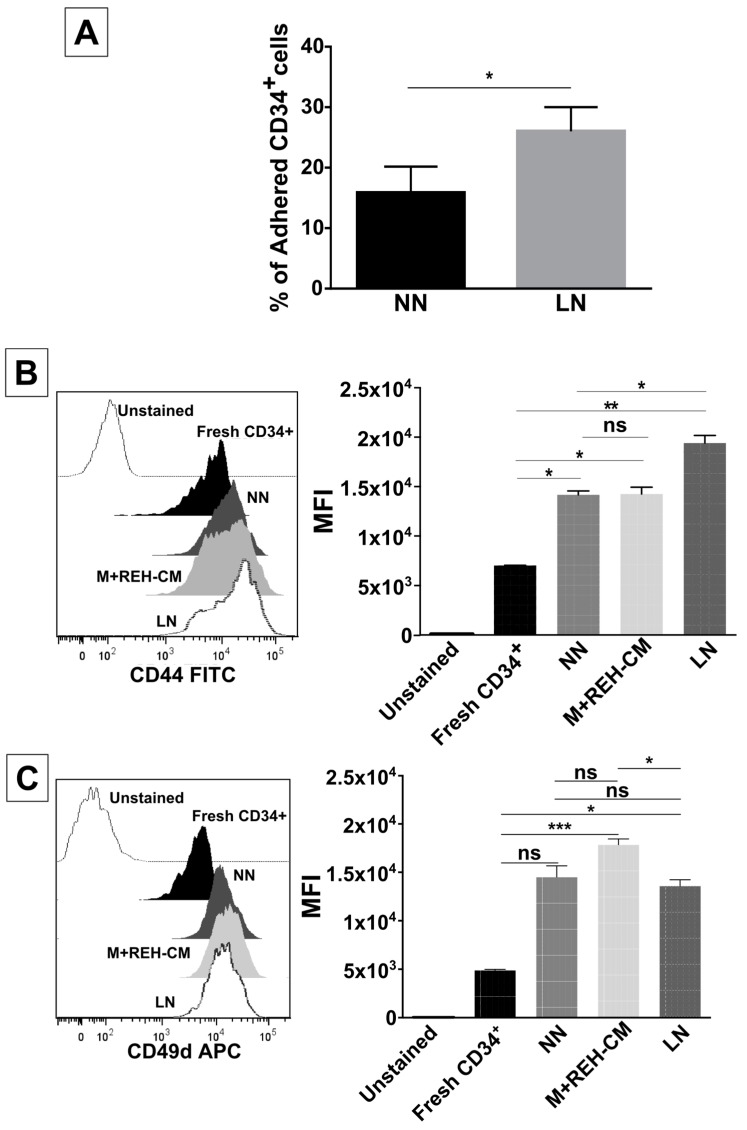

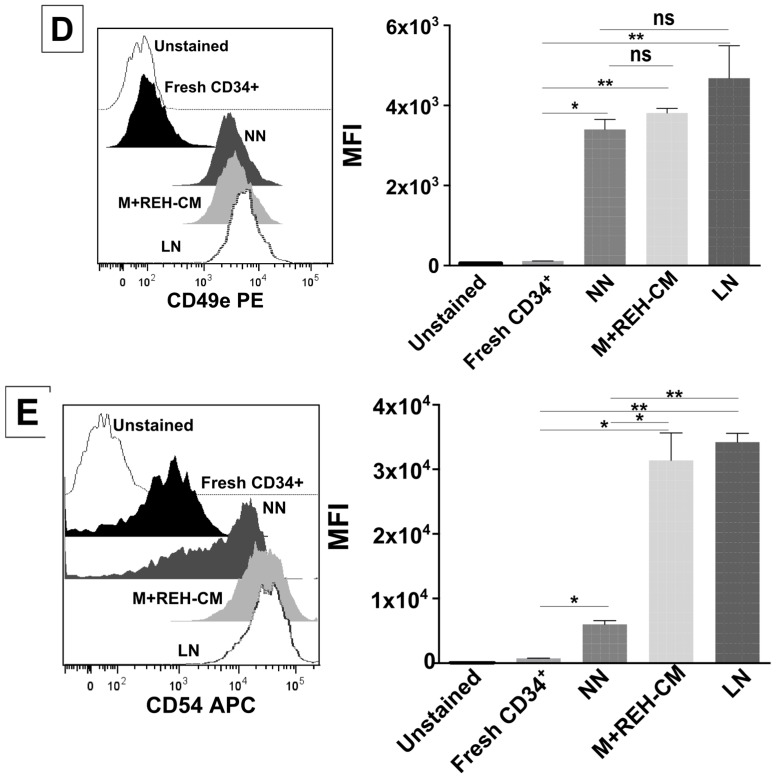
Increased adhesion capacity and expression of some adhesion molecules in CD34+ cells in the LN. (**A**) MSC adhesion capacity evaluation of CFSE-labelled CD34+ cells obtained from the NN or the LN; cells were cultured with MSC for 4 h and counted by fluorescence microscopy. The percentage of adhered cells was calculated taking into account the total input of CD34+ cells (* *p* < 0.05). Labelling of (**B**) CD44, (**C**) CD49d, (**D**) CD49e, and (**E**) CD54 in freshly-isolated, NN, M+REH-CM, and LN CD34+ cells. Results are expressed as the median fluorescence intensity (MFI) from two independent experiments done in triplicates (*n* = 6) (ns: non-significant, * *p* < 0.05, ** *p* < 0.01, *** *p* < 0.001).

We next proceeded to evaluate CD34+ cells adherence to MSC after incubation in the NN or the LN. CD34+ cells isolated from the LN showed significantly more adhesion to MSC (Figure 2A). Cell adhesion molecule expression (CD44, CD49d, CD49e, and CD54) in CD34+ cells was then evaluated (Figure 2B–E). Although all cell adhesion molecules tested were upregulated in all niches when compared to freshly-isolated cells, no differences in MFI of CD49d and CD49e expression between CD34+ cells obtained from the NN or the LN were found (Figure 2C,D). Only CD44 (slightly) and CD54 (highly) expressions were increased, in the LN compared to the NN (Figure 2B,E). It is notable that the increased expression in all adhesion molecules evaluated here could be effectively simulated by the LN established with the REH-CM (M+REH-CM) (Figure 2B–E). In particular, CD49d upregulation was higher in the M+REH-CM than in the LN (Figure 2C) and the higher CD54 upregulation in the CD34+ cells obtained in the LN was totally reproduced by the M+REH-CM.

**Figure 3:**
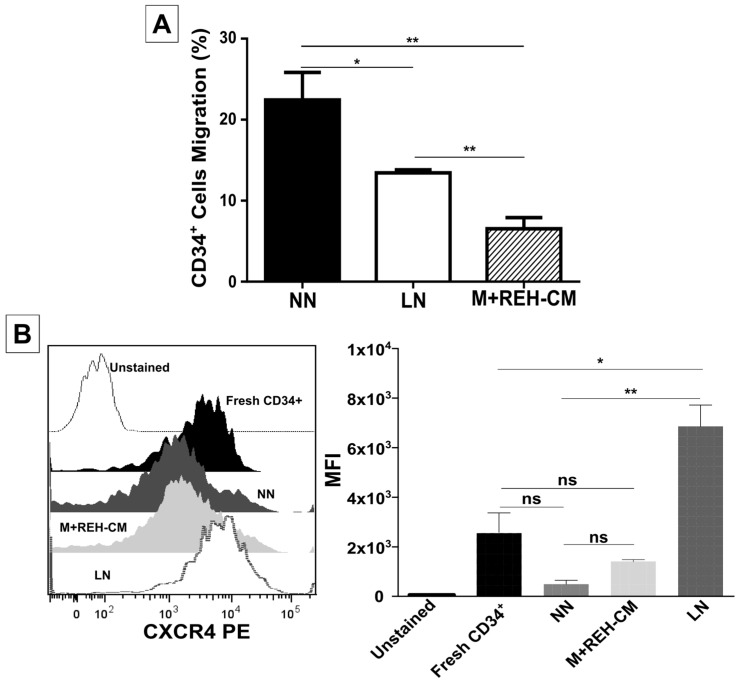
Decreased migration of CD34+ cells in a leukemic microenvironment. (**A**) The CD34+ cells migration capacity towards the chemoattractant SDF-1 was determined in a transwell system with a 5 μm pore membrane; CD34+ cells from the NN or the LN and M+REH-CM CD34+ cells were allowed to migrate for 4 h, after which cells in the lower chamber were harvested and counted by flow cytometry. The percentage of migration was calculated considering the total input of CD34+ cells; (**B**) Labelling of CXCR4 (CD184) in freshly-isolated cells, NN, M+REH-CM, and LN CD34+ cells. Results are expressed as the median fluorescence intensity (MFI) from two independent experiments done in duplicates (*n* = 4) (ns: non-significant, * *p* < 0.05, ** *p* < 0.01).

In agreement with a higher adhesion to MSC, CD34+ cells from the LN showed less SDF-1-directed migration (Figure 3A) compared to CD34+ cells from the NN. Interestingly, the M+REH-CM had a stronger inhibitory effect than the LN in cell migration (Figure 3A). Intriguingly, CXCR4 expression was higher in the LN and in the M+REH-CM compared to NN (Figure 3B), suggesting that CXCR4 activation and endocytosis is impaired in a leukemic context.

### 2.3. Primitive Markers Evaluation of CD34+ Cells in the LN

**Figure 4:**
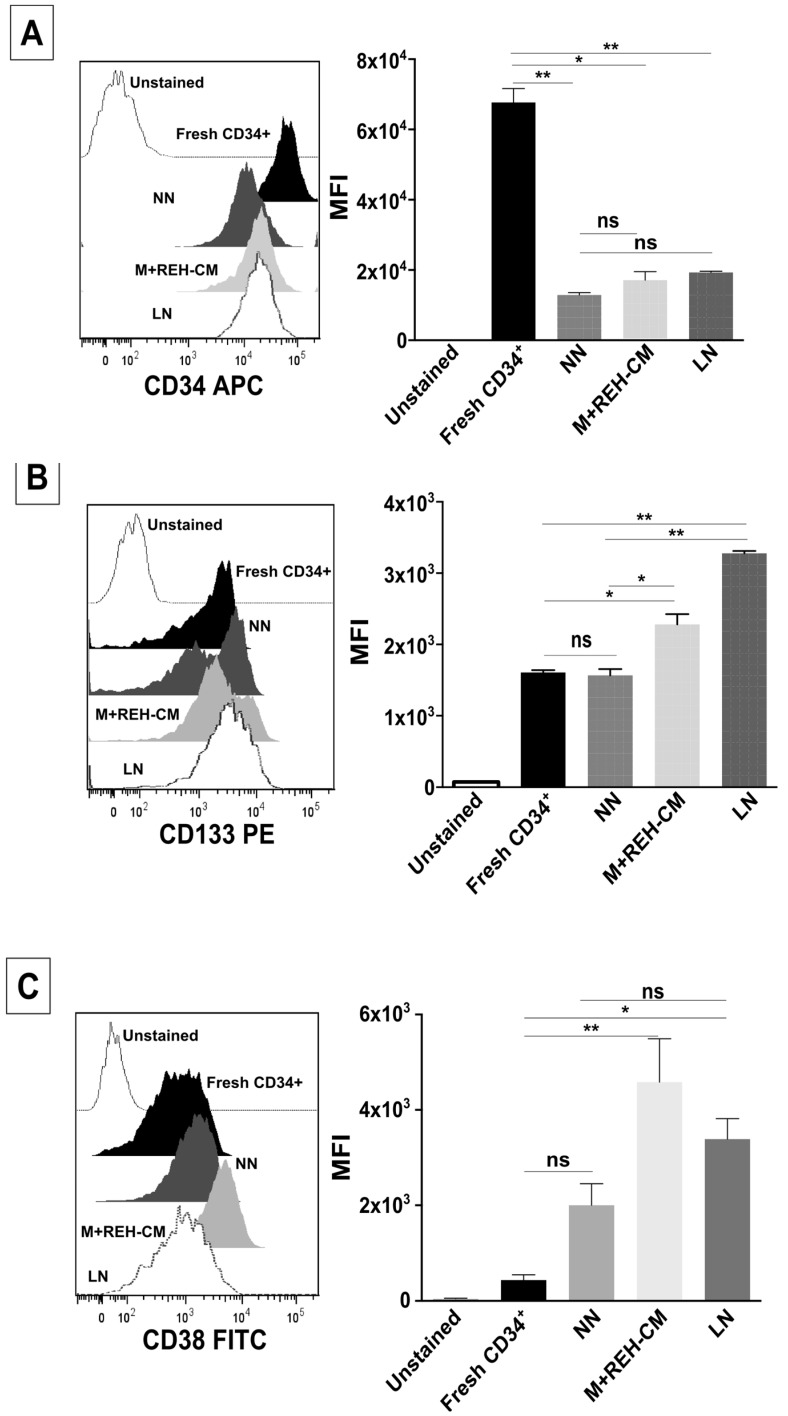

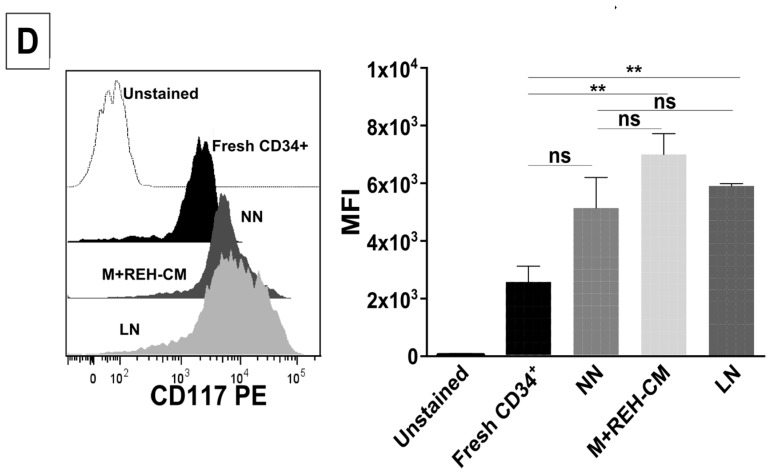
Changes in stem cell and differentiation markers of CD34^+^ cells in the LN. Flow cytometry analysis of (**A**) CD34, (**B**) CD133, (**C**) CD38, and (**D**) CD117 (c-Kit) expression in freshly-isolated, NN, M+REH-CM, and LN CD34^+^ cells. Results are expressed as the median fluorescence intensity (MFI) obtained from two independent experiments done in triplicates (*n* = 6) (ns: non-significant, * *p* < 0.05, ** *p* < 0.01).

The expression of the primitive marker CD34 was reduced in the NN, the M+REH-CM, and the LN compared to freshly-isolated cells (Figure 4A). When comparing the different niches, only a slightly increase in the LN was observed. Otherwise, CD133 was slightly upregulated in both leukemic microenvironments compared to the NN (Figure 4B) with the M+REH-CM having a lower effect than the LN. In freshly isolated CD34+ cells, CD133 expression was more variable. In the NN condition, two cell populations could be distinguished with middle and high CD133 expression, while in the LN only the high CD133 expressing population prevails. On the other hand, CD38 expression was low in fresh cells, increasing slightly only in the M+REH-CM (with no differences between the different niches) (Figure 4C). Compared to freshly-isolated cells, all niches induced a higher expression of CD117 (Figure 4D), with no differences in expression between cells from the NN and the LN or the M+REH-CM. These results showed that a primitive phenotype (CD34+, CD133+ and CD117+) tended to be maintained when comparing the NN and both LNs, although a tendency towards higher expression is evidenced in the LNs. The double-labeling of cells in the context of CD34 expression showed the maintenance of CD34+CD133+, CD34+CD38+, and CD34+CD117+ cell populations in all niches (Figure S4A–C).

### 2.4. Multipotency Determination in the LN

**Figure 5:**
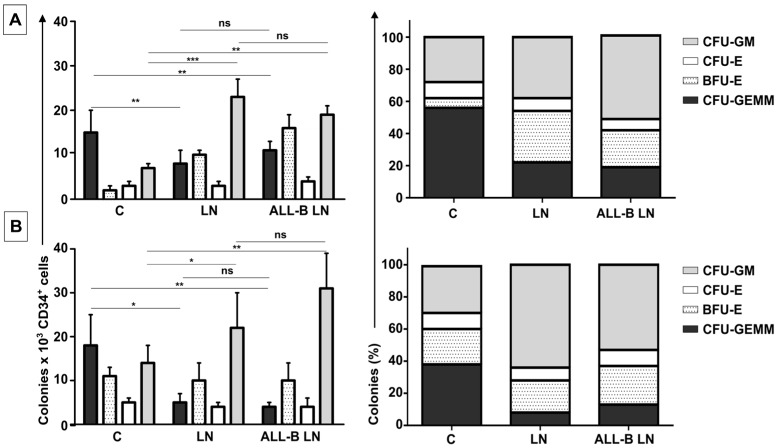
Reduced primitive colony forming units of CD34+ cells exposed to the LN. Colony-forming units of granulocyte/erythrocyte/monocyte/megakaryocyte (CFU-GEMM), burst forming unit erythroids (BFU-E), CFU-erythroids (CFU-E), and CFU-granulocyte/macrophage (CFU-GM) by CD34+ co-cultured in the NN or the LN (LN established with REH cells, middle columns; LN established with primary ALL-B cells, right columns); absolute (left panels) and relative counts (right panels) for two different CD34+ samples were performed in duplicate (*n* = 4), panels (**A**) and (**B**), are shown. Results are expressed as the mean ± SEM (*p*-values: non-parametric one-way ANOVA; ns: non-significant, * *p* < 0.05, ** *p* < 0.01, *** *p* < 0.001).

More importantly, we found that CD34+ cell multipotency was also significantly affected in the LN, with a significant decrease in the absolute count and percentage of primitive progenitor UFC-GEMM colonies (>50%) that was accompanied by an increase of other progenitor colonies, especially UFC-GM and BFU-E (Figure 5, middle columns labeled LN in all panels). Results were very similar for two different UCB samples used (Figure 5A,B).

### 2.5. CD34+ Cells Evaluation in a LN Established with Primary Cells from an ALL-B Patient

We were also interested to know whether the effect on CD34+ cells seen in this LN (with REH cells) could also be observed in a LN established with primary leukemic cells isolated from an ALL-B patient. It was important to obtain a BM sample from an ALL-B patient presenting high blast infiltration (>96%) for cell isolation. Due to limitations in the volume of the available sample, only few evaluations were performed. First, multipotency was evaluated (Figure 5A,B, right columns in all panels), with results equivalent to those obtained with the LN set with the REH cell line, i.e., an important reduction in primitive CFU-GEMM colonies (absolute count and percentage) in two different UCB samples. As before, with the REH LN setting, this reduction was accompanied by an increase in CFU-GM and BFU-E. It is of note that no major differences in progenitors were found in the LN set with the ALL-B patient cells or with the REH cell line.

**Figure 6:**
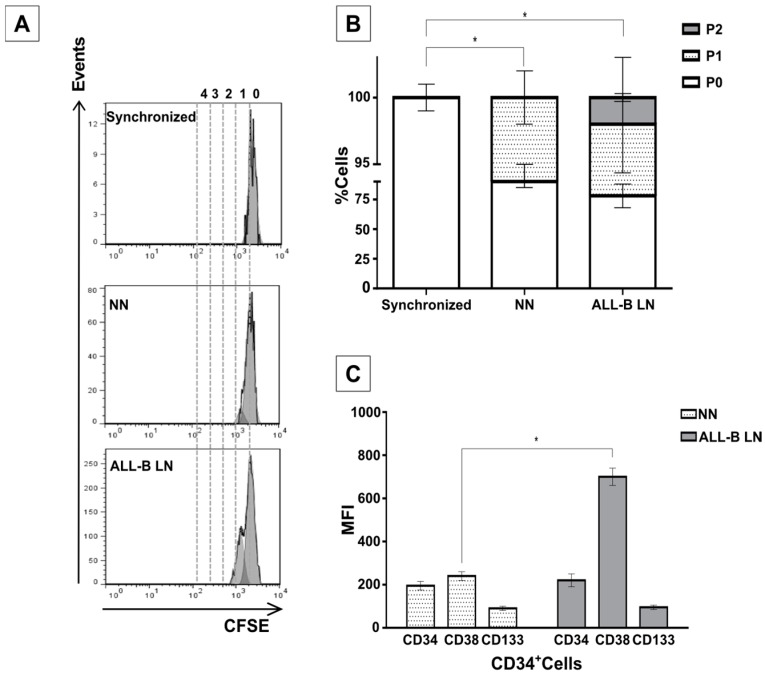
Increased proliferation and abnormal differentiation of CD34^+^ in a LN established with leukemic blasts from an ALL-B patient. (**A**) CD34+ cells isolated from an UCB sample and co-cultured with MSC pre-exposed to primary leukemic cells showed higher proliferation in the LN than in the NN. Synchronized cells are shown for comparison. A representative experiment is shown; (**B**) quantification of cells division in all settings are shown; and (**C**) mean fluorescence intensity (MFI) of the CD34, CD133 and CD38 markers in HSC co-cultured in the NN or in the ALL-B-LN. Data were obtained from two independent experiments done in duplicate (*n* = 4) (* *p* < 0.05).

CD34+ cell proliferation also increased when two different UCB samples were used in the LN established with cell blasts isolated from an ALL-B patient (Figure 6A,B). This result is very similar to the findings in the LN with the REH cell line (please compare with Figure 1A). Additionally, culturing MSC in the presence of ALL-B primary cells induces a CD38 upregulation that we did not observe in the M+REH-CM or in the LN (Figure 4C). No increase in the expression of the primitive markers CD34 and CD133 were observed (Figure 6C), again comparable to what we have described for the LN set with the REH cell line. Therefore, it seems that, except for some slight differences, the LN set with the REH cell line properly replicate the LN set with primary cells isolated from an ALL-B patient.

### 2.6. MSC, REH, and LN Supernatants Characterization

**Figure 7:**
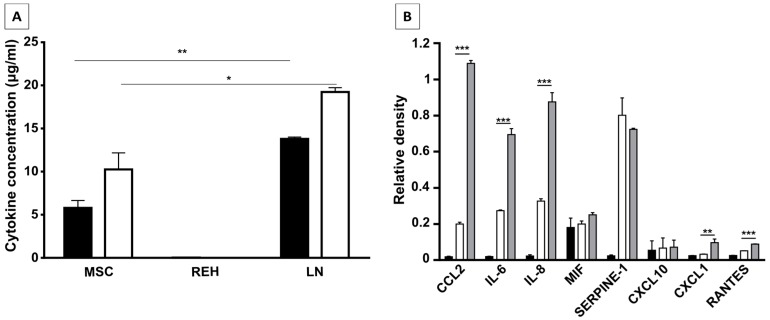
Identification and quantification of cytokines present in leukemic microenvironment. (**A**) IL-6 (white bar) and IL-8 (black bar) quantification in CM of MSC, REH, and LN. Quantification of cytokines was made by CBA (cytometric bead array, Human Inflammatory Cytokines Kit, BD) after 24 h of monocultures (MSC or REH) and co-cultures (LN) with 1% of FBS. (**B**) Cytokines present in the CM of REH cells (black bar), MSC (white bar), and LN (grey bar) were observed by microarray analysis. Relative densities are shown compared with positive control spots. Identification of cytokines present in LN was made by Proteome Profiler Array (Human Cytokine Array) R and D Systems. Data were obtained from the two independent experiments done in duplicates (*n* = 4) (* *p* < 0.05, ** *p* < 0.01, *** *p* < 0.001).

Given that the described abnormal phenotype and function of CD34+ cells were very similar in the LN and the M+REH-CM we were interested to know which soluble factors could be responsible for the observed effect. A CBA assay detecting six inflammatory cytokines showed initially that the LN was enriched in IL-6 and IL-8 (Figure 7A). A microarray assay including more than 36 soluble and growth factors showed again the great enrichment of IL-6 and IL-8 alongside CCL2 (Figure 7B) in the LN compared to MSC or REH cells alone. A small increase in CXCL1 and RANTES was also observed. The real contribution of each one of these cytokines to the observed effects deserves further work.

## 3. Discussion

Early work has shown that ALL-T cells modify hematopoietic stem cell growth both in vivo and in vitro [21]. More recently, CML and ALL mouse models have shown that a reduction in SDF-1 levels in BM is responsible for impaired LT-HSC or human CD34+ homing and retention [17,18,19,20]. This apparently modest change in cytokine production by stromal cells has profound effects on HSC biology. SDF-1 can function as an inhibitor of human LTC-IC cycling [22], and its reduction in BM contributes to increased cell cycling. Consistent with this, as we have shown here in our in vitro LN model, is that CD34+ cells proliferate more and have increased cell populations in the S/G_2_/M phases of the cell cycle when compared to CD34+ cells in the NN. An even higher cell proliferative response was observed when CD34+ cells were previously incubated with the REH-CM, signifying that soluble factors secreted by the leukemic cells are responsible for this effect. Nevertheless, the CD34+ cell proliferation rate was lower when compared to cells expanded in the presence of early cytokines (SCF, TPO, and Flt3L) and in the absence of MSC. These results showed that MSC previously co-cultured with leukemic cells or with REH-derived CM, partially (LN) or totally (REH-CM) lose their control over the CD34+ cell quiescence.

On the other hand, CD34+ cell binding capacity to MSC was enhanced in the LN. This was accompanied by an increased expression of CD44, CD49e, and CD54. Particularly, CD54 (ICAM-1) was highly upregulated in the LN compared to the NN, suggesting its involvement in CD34+ cell adhesion. The M+REH-CM showed the same effect as the LN on this adhesive phenotype, suggesting that soluble factors in the LN are responsible for these alterations. This situation is similar to the phenotype described for MSC from ALL patients having increased adhesion induced by soluble factors [23]. The specific molecules responsible for the higher cell adhesion to MSC should be further explored. As expected, this higher cell adhesion to MSC in the LN was accompanied by a reduced CD34+ cell migration to SDF-1. Higher CXCR4 expression was observed in CD34+ cell from the LN, suggestive of an inadequate endocytosis and altered CXCR4 signaling. On the contrary, a reduction in CXCR4 expression was evident in the NN, showing an appropriate SDF-1 signaling. The situation with the M+REH-CM was similar, with reduced cell migration and higher CXCR4 expression compared to NN. To our knowledge, the evaluation of CXCR4 expression in HSC in patients with leukemia has not been done. Nevertheless, it has been shown, in an experimentally-induced leukemia model, that HSC increased CXCR4 expression [24], showing the similarity to our in vitro model. Additionally, it has been shown that BM levels of SDF-1 in pediatric precursors of ALL-B were almost three times lower compared to non-leukemic controls [25]. As expected, we did not find SDF-1 secretion in the LN. Interestingly, it has been shown in leukemic mouse models that HSC respond better to SCF [20], and it was suggested that less retention in response to SDF-1 would facilitate displacement in response to this factor or others present in this in vivo setting.

This increased proliferation of CD34+ cells, accompanied by an augmented cell adhesion to MSC and a reduced migration capacity to SDF-1, could also alter the conventional signals arising from HSC-MSC interactions and indirectly affect other HSPC functions. In relation to the expression of primitive markers, it is interesting to point out that the expression of CD34 was similar in the NN and LN (with a slightly increase in the LN), although reduced compared to freshly-isolated cells. Additionally, CD133 was upregulated in the LN and in the M+REH-CM. CD117 (c-kit) expression has been used as an indication of a primitive status and multipotency capabilities [26]. Compared to freshly isolated cells, CD117 was slightly upregulated with practically no differences between NN, LN, and M+REH-CM. Our in vitro cultures induced a higher expression of the CD38 marker in the different niches compared to freshly isolated cells, but no statistical differences were found between the NN and the LN or M+REH-CM (although a tendency towards a higher expression was noticed in the latter). These results are similar to what has been described for leukemic patients and in a mouse model of AML [27]. They suggest that signals responsible for a primitive cell state are still present in the LN. They seem to be slightly incomplete for HSPC, but they are possibly suitable for the leukemic cells. To further explore this issue, the differentiation potential into hematopoietic lineages was also evaluated in cells obtained from both the NN and the LN. Here again, an important reduction in the absolute count and the percentage of primitive progenitor colonies (CFU-GEMM) was detected in cells obtained from the LN, accompanied by an increase of CFU-GM. These changes were also similar to cytokine-expanded CD34+ cells when compared to a similar setting of the NN [26]. This is also consistent with a study in a leukemia mouse model that showed a loss of HSC self-renewal capacity and differentiation [28]. No important differences were observed in erythroid precursors (BFU-E or CFU-E), a situation that has been described in CLL patients in spite of the occurrence of the disease-related anemia [29].

Remarkably, HSPC alterations were also observed when the leukemic microenvironment was established by incubation of MSC with primary leukemic cells isolated from an ALL-B patient. Increased proliferation, reduced clonogenic capacity, significant upregulation of CD38, and primitive marker (CD34 and CD133) expression maintenance were also confirmed in this system. This is a valuable finding, since further insight into the mechanisms involved in these interactions could be perfectly performed with the REH cell line. This would certainly facilitate further HSPC evaluation in this in vitro leukemic microenvironment.

Altogether, these results support the concept that the control imposed on stem cells and progenitors cells by normal MSC is lost or severely reduced in the LN. Paradoxically, in spite of a higher adherence to MSC, HSPC from the LN proliferate extensively, differentiate abnormally, and lose their clonogenic capacity. This is very similar to the findings described by Welner et al. [30], who demonstrated that CML caused normal mouse HSPC to divide more readily, altered their differentiation, and reduced their reconstitution and self-renewal potential.

Knowing that in normal circumstances both cell-cell interactions and soluble factors are responsible for HSC homeostasis, in our in vitro model of the LN it seems to be clear that the cytokines secreted in the LN, either by the REH cells, by the MSC co-cultured with REH cells (or both), play a major role in HSPC malfunction. In fact, most of the changes observed in the LN could be replicated by incubation of MSC with the REH-conditioned medium (M+REH-CM), suggesting that paracrine stimulation is responsible for the CD34+ cell alterations. The fact that some soluble factors (CCL2, IL-6, IL8, CXCL1, and RANTES) are synergistically increased in the LN argues in favor that the phenotypic and functional alterations observed in the CD34+ cells is either directly through soluble factors secreted from the REH cell and/or indirectly through REH-CM-induced secretion by MSC. In particular the role of CCL2, IL-6, and IL-8 merits further study; the fact that IL-8 and CCL2 are upregulated in MSC from ALL patients [23], and that plasma levels of CCL2, IL-8, and IL-6 are increased in children at ALL diagnosis [23,31], show their relevance in vivo and validates in part our in vitro LN. The leukemic-induced alterations of the HSPC found here could be equated with the observed reduced hematopoiesis, increased progenitors and differentiated cells, and, in general, bone marrow failure in patients with ALL [6,20,32]. On the other hand, we have shown here that signals responsible for maintaining a primitive phenotype persist in the LN, being certainly used by the leukemic cells for their own benefit. Finally, this in vitro system will allow us to understand the different functional consequences of these multiple interactions (cell–cell and cell-soluble factors) occurring in the LN, also producing valuable information related to the changes occurring in MSC and leukemic cells, with meaningful implications for novel therapeutic approaches.

## 4. Materials and Methods

### 4.1. BM Mesenchymal Stem Cell Isolation and Characterization

BM aspirates from the iliac crest of healthy donors aged 5–12 years were obtained after their parents had signed an informed consent and the ethical committees of the participant institutions had given approval. Samples were collected in a sterile tube containing 0.25% EDTA (GIBCO-Invitrogen, Grand Island, NY, USA), and mononuclear cells (MNC) were isolated by Ficoll density gradient centrifugation (Histopaque *d* = 1.077 g/cm^3^, Sigma-Aldrich, St. Louis, MO, USA). MNC were plated at a density of 10^6^ cells/cm^2^ in Iscove’s Modified Dulbecco’s Medium (IMDM) Glutamax-I (GIBCO-Life Technologies, Grand Island, NY, USA) supplemented with 1% sodium pyruvate (GIBCO-Life Technologies, Grand Island, NY, USA), 1% Minimum Essential Medium (MEM) non-essential amino acid solution 100X (GIBCO-Life Technologies, Grand Island, NY, USA), and 10% fetal bovine serum (FBS, GIBCO-Life Technologies, Grand Island, NY, USA). After obtaining 90% cell confluence, adherent cells were detached by treatment with 0.25% Trypsin (Sigma-Aldrich, St. Louis, MO, USA) and 1 mM EDTA. Cells were characterized by means of immunophenotyping and differentiation assays (see below) and were used for the different experiments in passages 3–5.

After the third passage, adherent cells were trypsinized and labeled with the following monoclonal antibodies: Fluorescein isothiocyanate (FITC) mouse anti-human CD73 (clone AD2, BD Pharmingen, San Jose, CA, USA), Allophycocyanin (APC) mouse anti-human CD105 (clone SN6, Invitrogen, Frederick, MD, USA), FITC mouse anti-human CD90 (clone F15-42-1, Abcam, Cambridge, MA, USA), and FITC anti-human CD44 (clone MEM-85, Invitrogen, Frederick, MD, USA). Additionally, the leucocyte-specific antibody PerCP mouse anti-human CD45 (clone 2D1, BD Biosciences, San Jose, CA, USA) and the APC mouse anti-human CD34 (clone 581, BD Pharmingen, San Jose, CA, USA) were used. Data were acquired using a FACSAria II flow cytometer (Becton Dickinson Biosciences, San Jose, CA, USA). FACS Diva software, CellQUEST PRO software, FlowJo, and Paint-A-Gate software (Pro v1.0, Becton Dickinson Biosciences, Sunnyvale, CA, USA) were used for data analysis.

Furthermore, the osteogenic, adipogenic, and chondrogenic differentiation capacities were determined using specific stainings and optical microscopy examination, as previously described [26]. Third-passage 2 × 10^4^ MSC were cultured in a 24-well plate in IMDM until they reached confluence. For adipogenic differentiation, cells were cultured for three days alternately in an induction medium (MEMα supplemented with 10% FBS, 1 mM dexamethasone, 0.5 mM isobutylmethylxanthine, 200 μM indomethacin, and 10 μg/mL insulin; all reagents were from Sigma Aldrich, St. Louis, MO, USA) or in a maintenance medium (MEMα, supplemented with 10% FBS and 10 μg/mL insulin) for two weeks. Osteogenic differentiation was induced by cell incubation in MEMα supplemented with 10% FBS, 100 nM dexamethasone, 0.2 mM ascorbic-2-phosphate, and 10 mM β-glycerophosphate (all reagents were from Sigma Aldrich, St. Louis, MO, USA) for two weeks. For chondrogenic differentiation, cells were plated and cultured in a chondrogenic induction medium (MEMα and 10 ng/mL TGFβ-1, Sigma Aldrich, St. Louis, MO, USA), also for two weeks. Then the cells were washed three times with PBS (1X), followed by fixation with formalin solution (Sigma Aldrich, St. Louis, MO, USA), and were stained with 0.35% Oil Red O solution (Sigma Aldrich, St. Louis, MO, USA) or alkaline phosphatase (using an AP staining kit, EMD Millipore Corporation, Billerica, MA, USA), or with 0.1% Safranin O (Sigma Aldrich, St. Louis, MO, USA). Cells were examined with an inverted microscope (Eclipse Model TS-100, Nikon, Konan, Minato-ku, Tokyo, Japan) and photographed with a Power Shot A460 Zoom Browser EX software (Canon, Melville, NY, USA).

### 4.2. CD34+ Isolation from Umbilical Cord Blood (UCB)

UCB samples from normal full-term deliveries were collected after obtaining informed consent and in accordance with the guidelines approved by the Ethics Committee of the Faculty of Medicine, Universidad Nacional de Colombia. MNC were isolated as described above, and CD34+ cells (HSPC) were purified through immunomagnetic selection using a MACS CD34+ isolation kit (Miltenyi Biotec, Auburn, CA, USA). Cell purity (>90%) was evaluated by flow cytometry using allophycocyanin (APC)-conjugated mouse anti-human CD34 antibody (Clone AC136, Miltenyi Biotec, Auburn, CA, USA), and cell viability (>95%) by trypan blue dye exclusion.

### 4.3. Establishment of a Leukemic Niche (LN)

The REH cell line was obtained from the American Type Culture Collection (ATCC; Rockville, MD, USA) and was characterized by flow cytometry for the presence of CD44, CD133, CD38, CD45, and CD19 (not shown). BM-MSC at 80% confluence were co-cultured for three days with the 3–5 × 10^4^ REH cells for the establishment of the leukemic niche (LN). After REH cells addition MSC stop dividing and did not increase cell number. After three days, the majority of REH cells were removed by gently pipetting with cold PBS (1X) and PBS plus 1 mM EDTA (1X). Freshly isolated CD34+ at a density of up to 100,000 cells/mL were added to cultured MSC-REH cells. In order to distinguish between the few REH cells remaining and the CD34+ cells, the latter were labeled in some experiments with 5 μM CFSE, as described below. The distinction between these two cell populations could also be achieved by CD44 or CD34 expression evaluation, since these cell surface markers are absent in the REH cell line. Simultaneously, freshly isolated CD34+ cells from the same UCB were layered over MSC that had reached 80% confluence, for the establishment of the so-called normal niche (NN). CD34+ cell evaluation in the NN or the LN was done after three days in the co-cultures. Some HSPC evaluations were also performed in a LN set by incubation of MSC with a REH-conditioned medium (M+REH-CM) without REH cells; in this case, freshly prepared REH-CM was added twice to cultured MSC for the establishment of the M+REH-CM leukemic microenvironment. For establishing the LN with primary leukemic cells (ALL-B-LN), we obtained BM mononuclear cells from a patient with ALL-B (having an infiltration of blasts in the BM > 96%) after signing the informed consent and we proceeded as described above for the LN.

### 4.4. REH-Conditioned Medium Preparation

2.5 × 10^5^ REH cells/mL were cultured in RPMI 1640 (Invitrogen Corporation, Carlsbad, CA, USA) supplemented with 1% sodium pyruvate, 1% MEM non-essential amino acid solution 100X and 1% FBS for 24 h at 37 °C and 5% CO_2_. Next, REH cells were centrifuged at 500× *g* for 7 min, and the medium was collected and filtered through a 0.22 µm pore membrane filter (Corning Incorporated Pittston, Pittston, PA, USA) and used fresh in all experiments.

### 4.5. CD34+ Cell Division Determination by Carboxyfluorescein Diacetate Succinimidyl Ester Labeling

CD34+ cells were labeled for 10 min at 37 °C with 5 μM carboxyfluorescein diacetate succinimidyl ester (CFSE; CellTrace™ CFSE Cell Proliferation Kit, Invitrogen, Eugene, OR, USA) in 0.1% bovine serum albumin (BSA, GIBCO-Invitrogen, Grand Island, NY, USA)-supplemented phosphate-buffered saline (PBS 1X). CFSE-labeled cells were suspended in RPMI 1640 (Invitrogen Corporation, Carlsbad, CA, USA) with 10% FBS for 5 min in ice and washed three times with PBS (1X) for 7 min at 400× *g* and 20 °C, resuspended in RPMI 1640 with 10% FBS, and further incubated 20 min at 37 °C. CFSE labeling was confirmed using fluorescence microscopy (Axiovert C-40 CFL, Carl Zeiss, Thornwood, NY, USA). A fraction of the CD34+ cells were FBS-deprived for 48 h (synchronized cells) to establish non-proliferating cells (the highest CFSE mean fluorescence intensity).

### 4.6. CD34+ Expansion Cultures

Purified and fresh CD34+ cells were incubated with the early cytokines TPO, SCF, and FLT3L (ProSpec, East Brunswick, NJ, USA) at a concentration of 50 ng/mL. After three days in expansion medium, cells were harvested and evaluated for cell proliferation.

### 4.7. Cell Cycle Analysis of CD34+

Purified and fresh CD34+ cells and CD34+ obtained from both the NN and the LN after co-culturing with MSC for the days were fixed with 4% formaldehyde and permeabilized with 0.1% Triton X-100 (Sigma Aldrich, St. Louis, MO, USA). Cells were incubated with 2′-(4-ethoxyphenyl)(-5-(4-methyl-1-piperazinyl)-2,5′-bi-1*H*-benzimidazole trihydrochloride trihydrate (Hoechst 33342, Invitrogen, Eugene, OR, USA) (2n DNA content, G_0_/G_1_; >2n DNA content, S-G_2_-M) for 45 min at 37 °C. Finally, the supernatants were removed and cells were resuspended in PBS (1X) for flow cytometry evaluation (FACSAria™ II, Becton Dickinson Biosciences, San Jose, CA, USA). FCS Express Flow Cytometry Data Analysis Software v5.0 (De Novo Software, Glendale, CA, USA) were used for data analysis.

### 4.8. CD34+ Cell Adhesion to MSC

For cell adhesion assays to MSC, 6 × 10^4^ MSC/mL were cultured for 24 h at 37 °C and 5% CO_2_ (80% of confluence). Next, 1 × 10^5^ CD34+ cells, previously stained with CFSE, obtained from both the NN and the LN, were co-cultured with MSC for 6 h. Non-adhered cells were carefully removed by two gentle washes with PBS (1X). Adherent cells were further incubated with warmed culture medium RPMI 1640 supplemented with 10% FBS, 1% sodium pyruvate, and 1% non-essential amino acid solution and counted in a fluorescence microscope.

### 4.9. Expression of Adhesion Molecules in CD34+ Cells after Co-Culture

CD34+ cells were harvested by repeated pipetting after three days in the niches, washed in PBS (1X), and stained with monoclonal antibodies for FACS analysis: APC-conjugated mouse anti-human CD49d (clone 9F10, BD Pharmingen, San Jose, CA, USA), PE-conjugated mouse anti-human CD49e (clone IIA1, BD Pharmingen, San Jose, CA, USA), FITC-conjugated mouse anti-human CD44 (clone MEM-85, Invitrogen, Frederick, MD, USA), and APC-conjugated mouse anti-human CD54 (clone REA266, Miltenyi Biotec, Auburn, CA, USA). Reliable discrimination between the few MSC present and CD34+ cells was possible using their different forward-scatter and side-scatter signals. Dead cells were excluded during acquisition and analysis (gate: intermediate forward-scatter and low side-scatter). FlowJo (v10.0, FlowJo, LLC, Ashland, OR, USA) and Paint-A-Gate software (Pro v1.0, Becton Dickinson Biosciences, Sunnyvale, CA, USA) were used for data analysis.

### 4.10. Expression of Primitive Markers in CD34+ after Co-Culture

CD34+ cells from NN or LN were washed in PBS (1X), and stained with monoclonal antibodies for FACS analysis: APC-conjugated mouse anti-human CD34 (clone AC136, Miltenyi Biotec, Auburn, CA, USA), PE-conjugated mouse anti-human CD133 (clone AC133, Miltenyi Biotec, Auburn, CA, USA), FITC-conjugated mouse anti-human CD38 (clone IB6, Miltenyi Biotec, Auburn, CA, USA), and PE-conjugated mouse anti-human CD117 (clone AC126, Miltenyi Biotec, Auburn, CA, USA), and analyzed as described above.

### 4.11. CD34+ Cell Clonogenic Capacity

Detection and quantification of human hematopoietic progenitors in the NN and the different settings of the LN were performed via colony-forming cell (CFC) assays. Cells (0.5–1 × 10^3^/1.1 mL) were cultured for 10–15 days in HSC-CFU, complete with EPO medium (StemMACS HSC-CFU Media, Miltenyi Biotec, Auburn, CA, USA). Colonies of erythroid progenitors (CFU-E and BFU-E), granulocyte-macrophage progenitors (CFU-GM, CFU-G, and CFU-M), and multi-potential granulocyte, erythroid, macrophage, and megakaryocyte progenitors (CFU-GEMM) were morphologically characterized and quantified by inverted microscopy, as described previously [26].

### 4.12. CD34+ Migration Assays

UCB-CD34+ cell migration assays were performed in Costar transwell (Corning Costar, Tewksbury, MA, USA), 6.5 mm diameter, and 5 µm pore size. Inserts were incubated for 1 h at 37 °C with the migration buffer (RPMI 1640/2% BSA). CD34+ cells (1 × 10^5^) isolated from the NN or the LN were added to the upper chamber. Lower chambers were filled with 600 µL of the migration buffer. For chemotaxis assays, human recombinant stromal cell-derived factor-1 (hrSDF-1a, Miltenyi Biotec, Auburn, CA, USA) at 100 ng/mL was added to the lower chamber. After 4 h at 37 °C and 5% CO_2_ incubation, cells that migrated to the lower chamber were collected and counted. Migrated cell percentage was calculated by dividing the number of migrated cells by the number of total input cells ×100. Percentages of migrated cells from at least four different CB samples were used for each paired analysis (FACS acquisition was performed in duplicate in some experiments).

### 4.13. Identification and Quantification of Cytokines

The concentration of six pro-inflammatory cytokines (IL-1β, TNF-α, IL-12-p70, IL-6, IL-8, and IL-10) present in the cell culture supernatants was determined using a human inflammatory cytokine kit BD™ Cytometric Bead Array (CBA) (BD Biosciences, San Jose, CA, USA) following instructions of the manufacturer. Supernatants were obtained from MSC cultured in supplemented IMDM, REH cells cultured in supplemented RPMI 1640, and co-cultures of MSC with REH cells, after three days of culture. Supernatants were centrifuged at 500× *g* for 7 min and filtered through a 0.22 µm pore membrane filter. A FACS flow cytometer was used to analyze samples. FlowJo and FCAP Array v3.0 Software (Becton Dickinson Biosciences, San Jose, CA, USA) were used for data analysis. Additionally, the Proteome Profiler Array/Human Cytokine Array (R&D Systems, Minneapolis, MN, USA), detecting more than 36 cytokines and growth factors, was used according to manufacturer’s instructions. Supernatants were obtained as above, except that they were collected after one day of culture.

### 4.14. Statistical Analysis

Cell proliferation, adhesion, and supernatant characterization data were analyzed using unpaired Student’s *t*-test. Cell cycle, cell surface, and primitive marker expression, migration, and colony forming unit data were analyzed using the Kruskal-Wallis test (non-parametric one-way ANOVA). The media comparison was made with Dunn’s multiple comparison test. GraphPad Prism 5.0 (GraphPad Software, Inc., La Jolla, CA, USA) was used for mathematical calculations and graphics. Results were considered significant when *p* < 0.05.

## 5. Conclusions

Using an in vitro model of a leukemic niche, we were able to show that HSPC proliferate extensively, differentiate abnormally and lose their clonogenic capacity, in spite of a higher adherence to MSC. This is similar to the findings in patients with acute lymphocytic leukemia who have defective hematopoiesis with increased progenitors and differentiated cells, and reduced reconstitution and self-renewal potentials. This in vitro leukemic niche could be a valuable tool for the understanding of the molecular events responsible for HSPC functional failure and a useful scenario for therapeutic evaluation.

## Supplementary Materials

Supplementary materials can be found at www.mdpi.com/1422-0067/18/2/199/s1.

## Abbreviations

MSC	Mesenchymal stem cells
HSPC	Hematopoietic stem and progenitor cells
SDF-1	Stromal cell-derived factor-1
ALL-B	Acute lymphocytic leukemia-B
BM	Bone marrow
HPC	Hematopoietic progenitor cells
HSC	Hematopoietic stem cells
CML	Chronic myeloid leukemia
MNC	Mononuclear cells
SCF	Stem cell factor
LN	Leukemic niche
NN	Normal niche
M+REH-CM	Leukemic niche set with REH-conditioned medium
REH-CM	REH-conditioned medium
UCB	Umbilical cord blood
CFSE	Carboxyfluorescein diacetate succinimidyl ester
TPO	Thrombopoietin
