# Supplementary material for: Phenotypic and Functional Alterations of Hematopoietic Stem and Progenitor Cells in an In Vitro Leukemia-Induced Microenvironment
Source: Int J Mol Sci. 2017 Feb 14;18(2):199. doi: 10.3390/ijms18020199 (PMC5343770; doi:10.3390/ijms18020199)
Supplement: Supplementary file 1 [file ijms-18-00199-s001.pdf]

Supplementary Materials: Phenotypic and Functional Alterations of Hematopoietic Stem and Progenitor Cells in an in Vitro Leukemia-Induced Microenvironment

Jean-Paul Vernot, Ximena Bonilla, Viviana Rodriguez-Pardo and Natalia-Del Pilar Vanegas

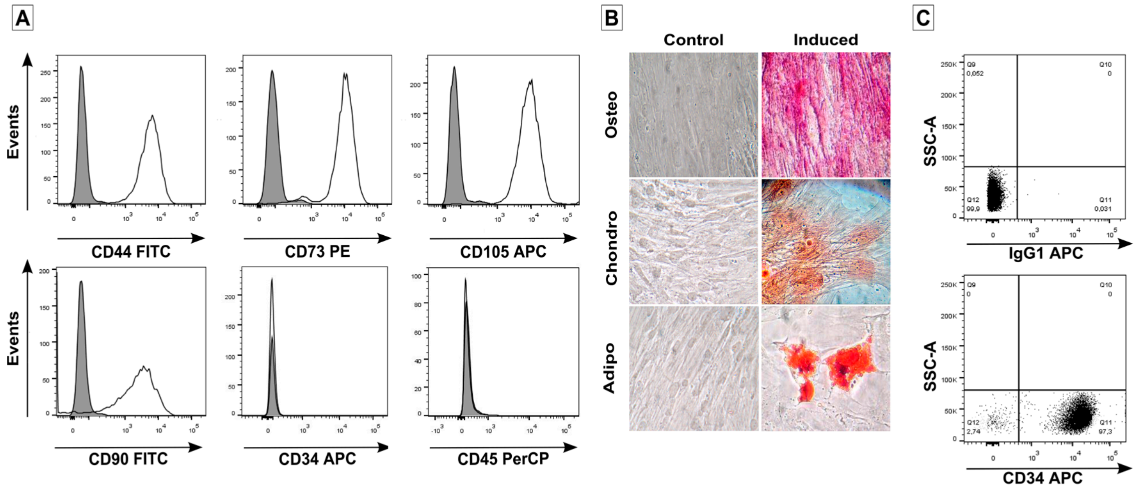

Figure S1. Isolation and characterization of MSC and CD34+ cells. (**A**) BM-MSC immunophenotype characterization by flow cytometry (grey histograms: isotype controls; antigen expression: white histograms). Cells were negative for PerCP mouse anti-human CD45 and APC mouse anti-human CD34 antibodies labeling; (**B**) differentiation potential of BM-MSC; Osteo: osteogenic differentiation detected by ALP staining (magnification 40×); Chondro: chondrogenic differentiation evidenced by Safranin O staining (magnification 40×); Adipo: adipogenic differentiation showing lipid-rich vacuoles stained with Oil Red O (magnification 40×). Left panels show control cells without differentiation induction. Photomicrographs were taken with a Canon PowerShot A640 camera adapted to an inverted microscope (Axiovert C40, Carl Zeiss, Thornwood, NY, USA). (**C**) Flow cytometry of purified CD34+ cells labelled with the isotype control (upper panel) and anti-CD34 APC (lower panel) monoclonal antibodies. Results shown are representative of one BM-MSC and one UCB sample.

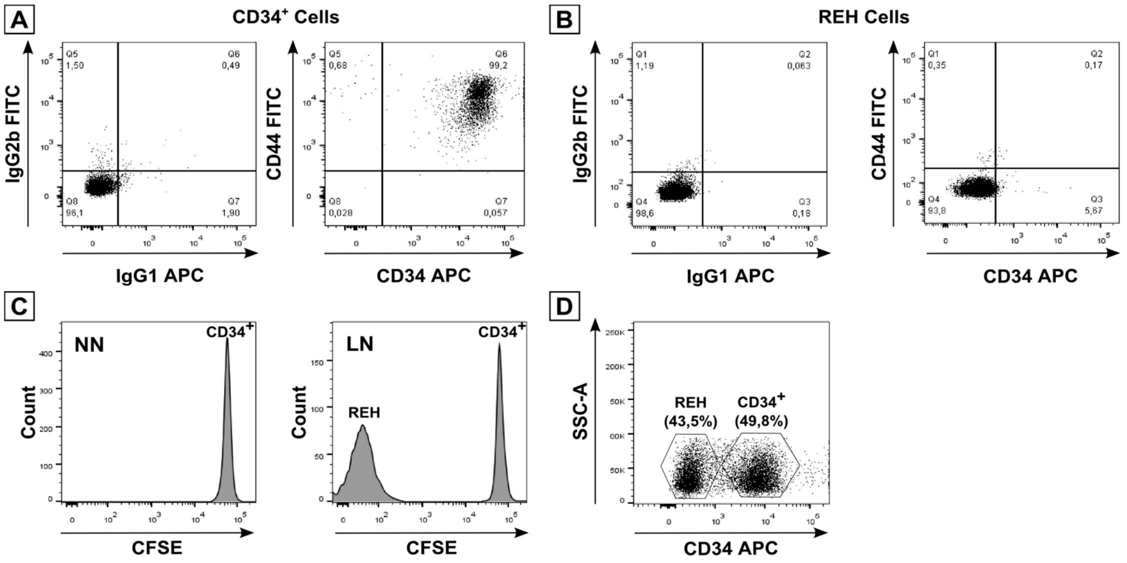

Figure S2. Cellular characterization in the NN and the LN. Characterization of CD34+ cells (**A**) and REH cells (**B**) by the expression of CD34 and CD44 surface markers. Isotype controls (left panels) and antigen expression (right panels). HSC were positive for these two markers while REH cells were negative; (**C**) flow cytometry analysis of CFSE-labelled CD34+ cells in NN and LN conditions; (**D**) percentage of CD34+ and REH cells present in the LN; the quantification was made by CD34+ staining and flow cytometry analysis. A representative experiment is shown.

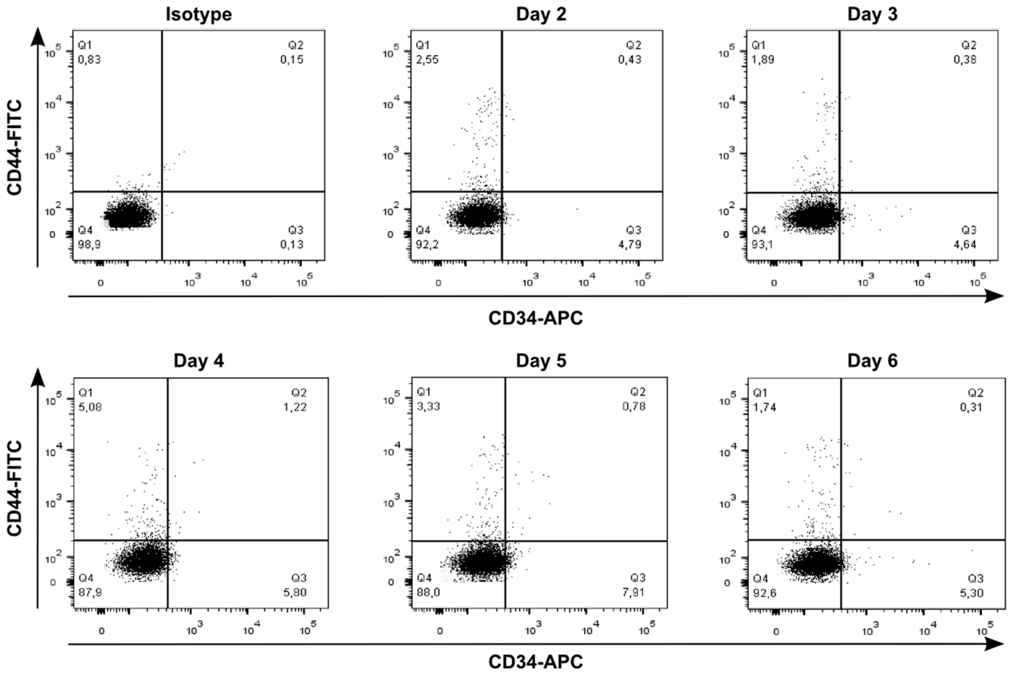

Figure S3. Evaluation of CD34 and CD44 expression in leukemic cells after culturing with MSC. CD34 and CD44 expression were evaluated in REH cells daily for six days in co-cultures with MSC. Analysis was done by flow cytometry. A representative experiment is shown.

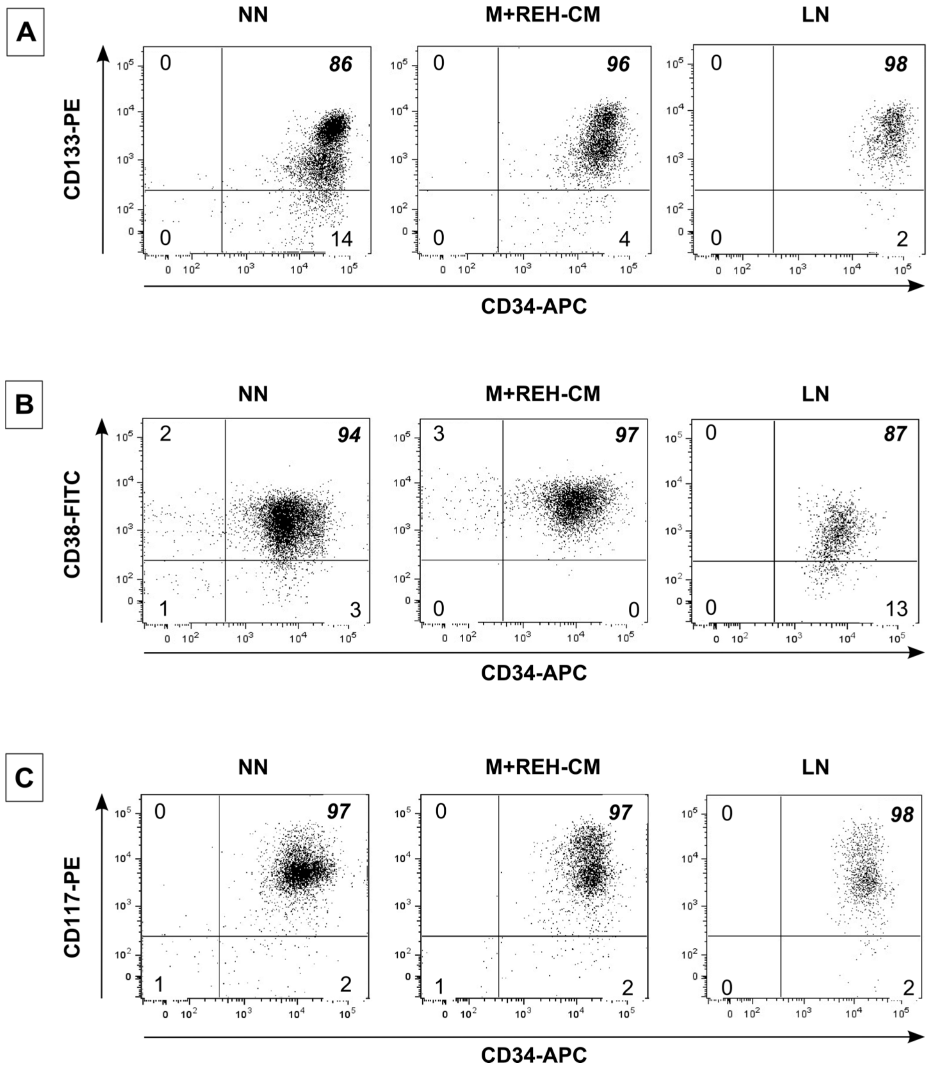

Figure S4. Flow cytometry analysis of double-labeled CD34+ cells after the co-culture in the NN, the LN, or the M + REH-CM. Dot plot of (**A**) CD34-APC vs CD133-PE (**B**) CD34-APC vs. CD38-FITC (**C**) CD34-APC vs. CD117-PE in NN, M + REH-CM and LN. Values within boxes represent the percentages of the live-gated cells. A representative experiment is shown.
